# Supplementary material for: Comprehensive Annotation and Functional Exploration of MicroRNAs in Lettuce
Source: Front Plant Sci. 2021 Dec 24;12:781836. doi: 10.3389/fpls.2021.781836 (PMC8739914; doi:10.3389/fpls.2021.781836)
Supplement: Supplementary file 3 [file Data_Sheet_9.ZIP › GSM2230751.plot/Lsa-miRN1696_Lsat_1_v5_gn_7_46921.1_773_TPlot.pdf]

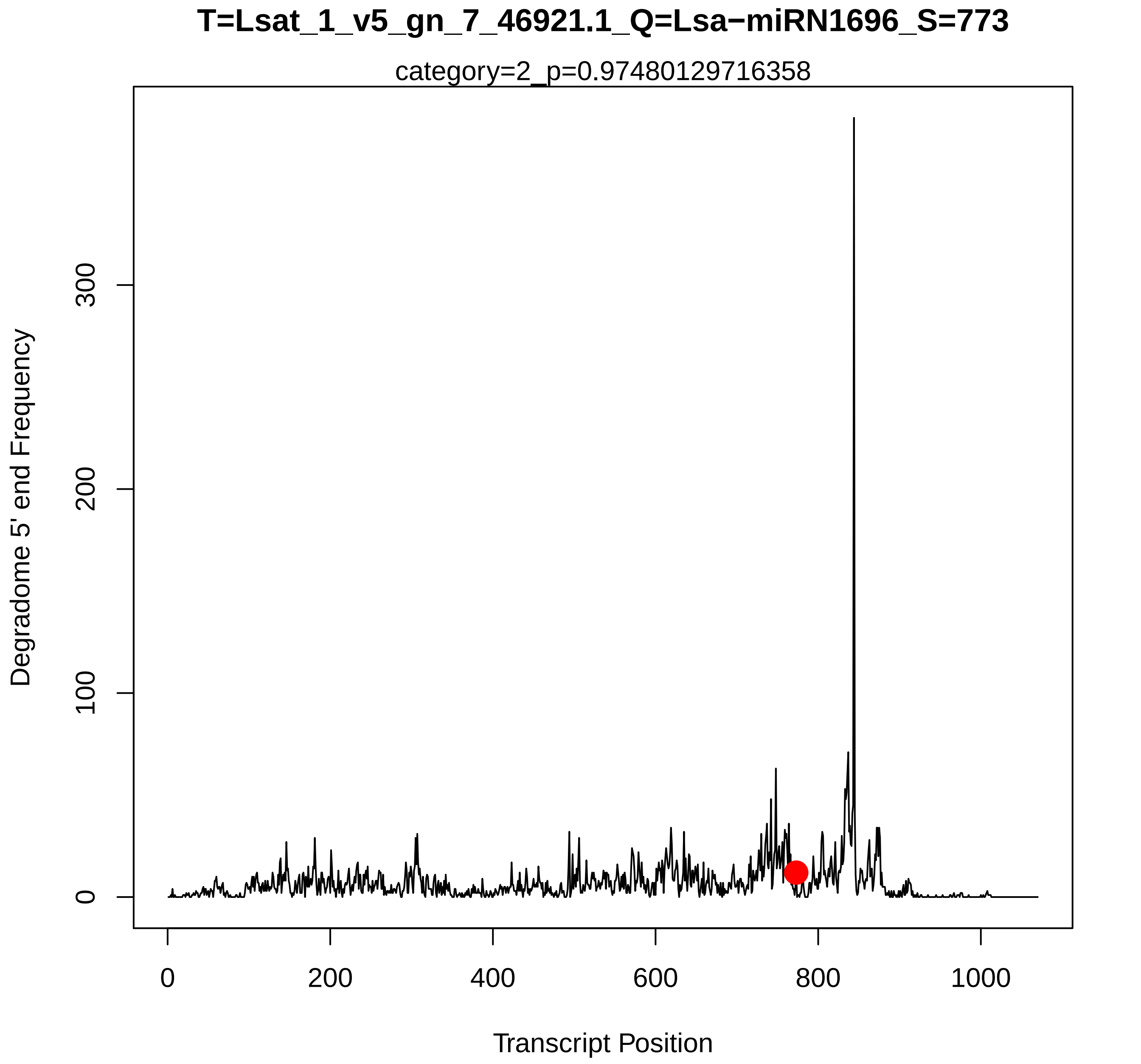
T=Lsat_1_v5_gn_7_46921.1_Q=Lsa−miRN1696_S=773
category=2_p=0.97480129716358
Degradome 5' end Frequency
0
100
200
300
0
200
400
600
800
1000
Transcript Position
